# Bioaugmentation Technology for Treatment of Toxic and Refractory Organic Waste Water Based on Artificial Intelligence

**DOI:** 10.3389/fbioe.2021.696166

**Published:** 2021-07-02

**Authors:** Jiang Yanbo, Jiang Jianyi, Wei Xiandong, Ling Wei, Jiang Lincheng

**Affiliations:** ^1^Research Center of Wastewater Engineering Treatment and Resource Recovery, Guangxi Beitou Environmental Protection and Water Group, Nanning, China; ^2^Institute of Ecological Engineering, Guangxi University, Nanning, China; ^3^Guangxi Civil and Environment Co., Ltd., Nanning, China

**Keywords:** artificial intelligence, intelligent control, waste water treatment, biofortification, fuzzy neural network

## Abstract

With the development of modern chemical synthesis technology, toxic and harmful compounds increase sharply. In order to improve the removal efficiency of refractory organic matter in waste water, the method of adding powdered activated carbon (PAC) to the system for adsorption was adopted. Through the analysis of organic matter removal rule before and after waste water treatment, it can be found that PAC is easy to adsorb hydrophobic organic matter, while activated sludge is easy to remove hydrophilic and weakly hydrophobic neutral organic matter. Powdered activated carbon-activated sludge SBR system (PAC-AS) system is obviously superior to AS and PAC system in removing organic matter of hydrophilic and hydrophobic components, that is, biodegradation and PAC adsorption are additive. Compared with the control system, the Chemical Oxygen Demand (COD) removal rate of refractory substances increased by 8.36%, and PAC had a good adsorption effect on small molecular weight organic compounds, but with the increase of molecular weight of organic compounds, the adsorption effect of PAC gradually weakened, and it had no adsorption effect on macromolecular organic compounds. Based on the research of fuzzy control theory, an Agent control system for ozone oxidation process of industrial waste water based on Mobile Agent Server (MAS) theory was established, which was realized by fuzzy control method. The simulation results showed strong stability and verified the feasibility and adaptability of the distributed intelligent waste water treatment system based on MAS theory in the actual control process.

## Introduction

In recent years, with the rapid development of economy, a large number of domestic industrial parks have emerged. The emergence of various industrial parks has caused a large amount of industrial waste water discharge, while most of the industrial parks in China are chemical industrial parks (Wu et al., 2018). Chemical enterprises produce various chemical industrial waste water in their production activities, the main sources of which are as follows (Boonnorat et al., 2018; Zhang et al., 2018): (1) raw materials for production; (2) By-products discharged in the production process; (3) Part of the final products in the production process; and (4) The waste water produced in the above production process due to transportation, washing, rain erosion, or other accidental factors, such as leakage. The waste water produced by various chemical enterprises is discharged and collected into comprehensive chemical waste water through the pipeline network. Therefore, comprehensive chemical waste water usually contains a large number of chemical inorganic or organic pollutants.

In recent decades, a large number of synthetic compounds have entered the environment. Due to their structural complexity and biological strangeness, it is difficult for them to be utilized by microorganisms and enter the material circulation in a short time. Refractory organic compounds are the main components of these compounds, and their treatment challenges the original treatment facilities and technologies. Aniline is an important chemical raw material, which is widely used in national defense, printing and dyeing, plastics, pesticides and pharmaceutical industries. At the same time, it is also a harmful substance that seriously pollutes the environment and endangers human health. Due to the toxicity of aniline to ecological organisms, aniline has been listed in the “blacklist of China’s environmental priority pollutants,” which requires strict control in industrial drainage. At present, the treatment of aniline waste water at home and abroad mainly includes physical, chemical and biological methods (Fan et al., 2017; Ke et al., 2018), and the research results in recent years show that biological enhanced treatment is one of the important ways to eliminate amine in the environment (Weiwei et al., 2017; Santos and Daniel, 2020). Bioaugmentation technology can effectively remove target pollutants (Weiwei et al., 2019), accelerate system startup (Abbaslou et al., 2018), improve the system’s ability to resist hydraulic and organic loads (Jasper et al., 2017), and enhance the stability of system flora structure and function (Ying et al., 2018).

In industrial production, a considerable number of complex control processes are still dominated by manual operation and control, such as the control of some nonlinear objects with large time delay. In this way, not only the technology is imperfect, but also the error in operation is large, and the data detection cannot achieve the expected effect. The control object of waste water treatment system is also nonlinear and unstable, which limits the treatment efficiency and results of waste water treatment process. Artificial intelligence has gradually become an independent branch (Wang et al., 2018; Hong et al., 2020). In this paper, Mobile Agent Server (MAS) theory is applied to the field of waste water treatment combined with bioaugmentation technology, and a distributed waste water treatment intelligent system based on Multi-Agent is established, so that agents can run in parallel and cooperate with each other in distributed environment, and comprehensively realize the functions of measurement and analysis, intelligent control and fault diagnosis in waste water treatment process from the system point of view. Multi-level and multi-sided integrated research is not only beneficial to cross-infiltration among disciplines, but also of great significance to solve the problems faced by waste water treatment process control and keep up with the development of the frontier disciplines.

## The Innovation of this Research

In this paper, the water quality and biodegradability of comprehensive chemical waste water in a chemical industry park are deeply analyzed, the treatment effect of conventional biological process on toxic and refractory organic waste water is evaluated, and feasible strengthening schemes are put forward. The removal effect and removal mechanism of the system after the implementation of each strengthening scheme are investigated, which provides theoretical guidance and technical support for practical sewage treatment plants to treat this kind of refractory comprehensive chemical waste water.

## Research Status at Home and Abroad

### Research Status of Bioaugmentation Technology in Waste Water Treatment

Bioaugmentation technology is to enhance biomass by adding microorganisms with special functions to natural flora, so as to enhance the response of biomass to a specific environment or special pollutants. The effects between the input strains and the sediment mainly include:

#### Direct Action of High-Efficiency Degrading Bacteria

The most common way to apply bioaugmentation technology is to directly add microorganisms with specific degradation ability to target pollutants. In this mechanism of action, firstly, one or more efficient microbial strains with high tolerance and degradation and transformation ability, which use one or a certain kind of pollutants as the main carbon source and energy, need to be obtained through domestication, screening, enrichment, separation, mutagenesis and gene recombination. After repeated cultivation and domestication, the microbial population grows continuously, and finally, it is put into the waste water treatment system that needs targeted degradation or treatment.

#### Co-metabolism of Microorganisms

Co-metabolism of microorganisms means that some organic matters can not be oxidized as carbon source or energy source of microorganisms, but only when the growth substrate exists can microorganisms biodegrade or oxidize the non-growth substrate. Literature (Singh et al., 2021) found that the coexistence of o-dichlorobenzene and m-dichlorobenzene was beneficial to the degradation of chlorinated aromatic hydrocarbons in the whole system. The research results of Xiaojian et al. also show that in anaerobic treatment system, glucose and other easily degradable organic compounds as co-metabolism matrix can obviously improve the degradation effect of biphenyl.

Literature (Park and Oh, 2020) using SBR process to treat landfill leachate, the removal effects of COD, ammonia nitrogen, chroma and total dissolved solids have been significantly improved after adding activated carbon into the system; Literature (Nath et al., 2018) used traditional activated sludge process to treat pharmaceutical waste water. By comparing the treatment effect of the system before and after adding activated carbon, the results showed that the treatment effect of the system after adding activated carbon was continuously improved, and antibiotic pollutants were better removed. Literature (Atashgahi et al., 2018) compares biological system with activated carbon, simple biological system and activated carbon system to treat waste water containing Cr. The results show that the removal effect of Cr is obviously improved after adding activated carbon.

One of the important benefits of bioaugmentation is that it can be processed on demand. Studies have shown that under the condition of limited space, direct inoculation of microorganisms can provide an immediate solution for a series of failed treatment systems without adding equipment or adding less equipment, which can maximize the operation of the treatment system and enhance the degradation ability of the system to organic pollutants (Rodriguez-Rodriguez et al., 2017), and may also be the only way to ensure closed circulation and zero emission of waste water without stopping production (Tigini et al., 2018).

### Application Status of Artificial Intelligence Technology in Waste Water Treatment System

Artificial Intelligence (AI) is a discipline that simulates human intelligence. Since the rapid development of AI theory and technology in 1970s, foreign scholars began to explore the application of AI technology in waste water treatment field, in order to solve the key problems that depend on knowledge highly and determine the operation control effect of waste water treatment. After decades of development, many effective research results have been obtained.

Among many intelligent control methods, fuzzy control, expert control and neural network control are the three most typical methods.

At present, the application of fuzzy control in China mainly focuses on the design of lower-level fuzzy controller, while foreign countries have many successful experiences in upper-level and lower-level applications, and pay more attention to upper-level reasoning and decision-making applications. For example, literature (Boonnorat et al., 2018) proposed that six variables, such as effluent substrate concentration, effluent SS, MLSS, effluent ammonia nitrogen concentration, DO concentration and sludge discharge rate, were used in the upper reasoning of the control system, and the set values of DO, sludge reflux ratio and sludge discharge were given by fuzzy decision-making, which were implemented by the bottom control. Literature (Correa and Maranho, 2021) developed a simple, reliable, cheap and PLC-embedded fuzzy controller for waste water treatment. After fuzzification of input variables, the output of the controller was calculated by inference engine, and then the control quantity was obtained after anti-fuzzification.

From the current application point of view, there are few reports on the application of expert control in the field of waste water treatment and control in China, and expert systems are used more in the upper layer of control system in foreign countries to carry out reasoning and optimization calculation and generate the set value of the controlled quantity; The lower layer uses switch control, PID control, fuzzy control and other methods to control the set value. For example, in reference (Poddar et al., 2020), the expert system is used to monitor and diagnose the anaerobic treatment process of sewage; Document (Aparicio et al., 2020; Martinez-Gallardo et al., 2020; Raimondo et al., 2020) uses rule-based expert system to calculate the set value of DO according to influent flow rate and influent COD.

From the current application point of view, in the waste water treatment process control, ANN control mostly acts as a controller directly. For example, the real-time control of continuous flow SBR process was studied by using BP neural network to monitor ORP and pH on line in document (Tigini et al., 2019). The results show that BP network can not only accurately predict information for real-time process control, but also reduce hydraulic retention time and aeration energy consumption compared with fixed-time control, and at the same time improve total nitrogen removal rate and denitrification effect.

## Research Technique

### Waste Water Treatment Control System Based on MAS

#### Distributed Control Design Idea

The problems to be solved by distributed control system are the coordination and scheduling among control flow systems, the synchronization of process data and the maintenance of process status in distributed control system because the control subsystems are distributed on different devices (Yue et al., 2018). The reason why the distributed control system is more complex than the centralized control flow system is that it has many nodes, and there may be various errors or anomalies in the process of coordinating various control flow subsystems to work together. The sources of these problems can be roughly divided into: 1) the problems of the control management system itself; 2) External factors, including human error, network or hardware abnormality. Designing a robust step-by-step control framework can deal with errors and recover from them, thus ensuring that the control process is not interrupted.

#### Mobile Agent Server Structure of Distributed Control System

**FIGURE 1:**
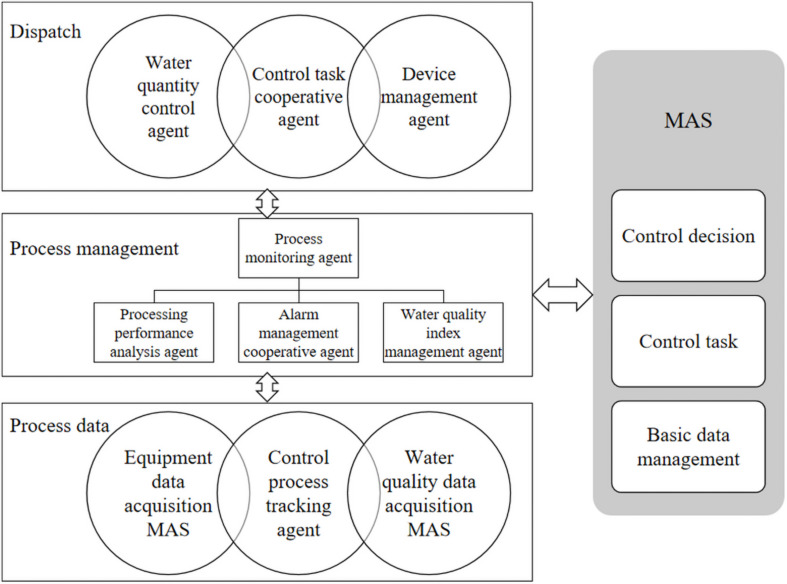
Mobile Agent Server (MAS) structure of distributed control system.

Considering the initiative and mobility of Agent technology, according to the specific requirements of control tasks, the control MAS (Mobile Agent Server) system structure is constructed, as shown in Figure 1.

Among them, the functions of the control task cooperative Agent include: receiving the control plan of the control plan MAS and transforming it into a control task list; Maintain task list information, including the number, quantity and start time of controlled equipment, track the execution process, and send status information to the control decision MAS in real time. The functions of standby management Agent include: Store the control parameters and usage amount representing the control system, and maintain the basic information of equipment, including equipment functions, attributes, capability levels, subordinate units, historical records, use depreciation, etc.; After the equipment obtains the task control list information, record the task information that the equipment needs to complete; When the equipment status changes, the equipment Agent can obtain the real-time information of the equipment operation through the basic data management MAS interaction, and automatically update the data.

#### Operation Model of Distributed Control System MAS

**FIGURE 2:**
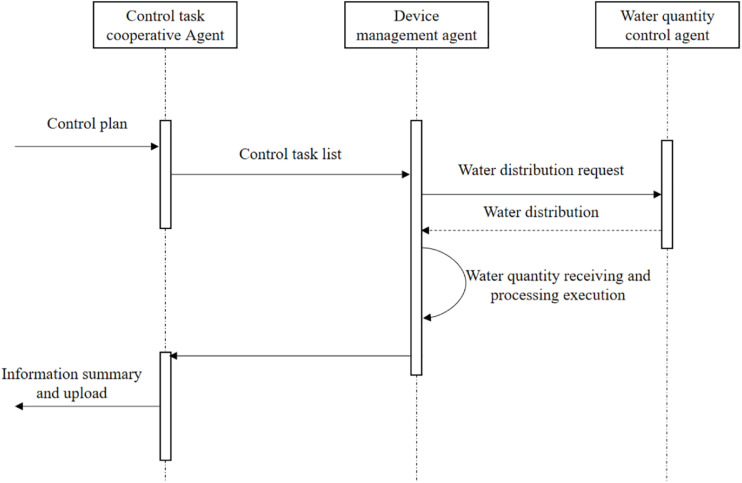
Information processing flow of MAS task dispatching in distributed control system.

Distributed control MAS can realize three functions: collaborative dispatching of control tasks, management of processing equipment Agent real-time data collection through multi-agent running process. In the process of control task collaboration, the control task collaboration Agent receives the control plan of the control decision MAS, sends a control task list and sends it to the equipment management Agent. At the same time, the water quantity distribution request is sent to the water quantity control Agent, which distributes the sewage to be treated. The equipment management Agent performs the operation tasks such as starting, stopping and numerical control adjustment of the control equipment, and feeds back the task execution. The interaction flow between Agent is shown in Figure 2.

### Fuzzy Neural Network Control System Model

**FIGURE 3:**
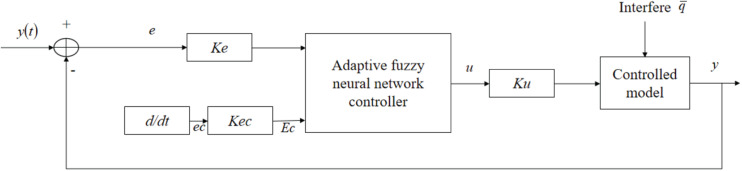
Fuzzy neural network control simulation system.

The structure of fuzzy neural network control system combined with waste water treatment system is shown in Figure 3. The nonlinear state equation obtained by the following formula is taken as the controlled model, and the adaptive fuzzy neural network controller optimized by the above parameters is applied to the system structure.

According to the law of material balance, based on the first and second equations of Lawrence-McCarty model and Mckinney model, the following nonlinear state equation of waste water treatment system is obtained through analysis and calculation (Changotra et al., 2019; Wang and Chen, 2020):

(1) $$\left\{ \begin{array}{c} \frac{\text{d}\,O}{\text{d}\,t}=0.57\,\lambda \,\left(S_{o},S_{e}\right)+1.1\,K_{e}\,X+\mu \\ \frac{\text{d}\,S}{\text{d}\,t}=\frac{K\,S\,X}{K_{s}+S}\\ \frac{\text{d}\,X}{\text{d}\,t}=\eta \,\frac{K\,S\,X}{K_{s}+S}-K_{d}\,X \end{array}\right.$$

In which:

*S*–aeration tank volume, m^3^;
η–the real yield of activated sludge microorganisms is obtained according to the empirical value of waste water treatment plant;
*K*_e_–endogenous respiration rate constant, h^−1^;
*K*–maximum specific substrate concentration utilization rate, constant, d^−1^;
*K*_s_–it is called half speed constant, and its value is equal to the substrate concentration at the maximum specific growth rate of half of microorganisms, mg/L;
λ–the reciprocal of water inflow time can be given according to the actual situation of waste water treatment plant, h^−1^;
*K*_d_–microbial attenuation constant of activated sludge, constant, d^−1^;
μ –air flow in aeration tank, m^3^/h;
*O*–concentration of dissolved oxygen in aeration tank, mg/L.

In the figure:

*e*,*ec*–error and error rate of change;
*Ke*,*Kec*–quantization factors input by the controller, respectively;
*E*,*Ec*–respectively, expressed as the error and error change rate after system quantization;
*Ku*–the quantization factor expressed as the output of the controller;
*y*(*t*)–expressed as the concentration of a given dissolved oxygen;
*u*–expressed as controller output;
*y*–expressed as the actual output dissolved oxygen concentration.

There is the following formula for calculating *Ke*,*Kec*,*Ku*:

(2) $$\begin{array}{c} K\,e=\frac{n}{e}\\ K\,e\,c=\frac{n}{e\,c}\\ K\,u=\frac{u}{n} \end{array}$$

Set the physical domain:

*N* = [−*n*, *n* + 1, ⋯, −1,0,1⋯,*n*],

*n* = 6, *e* = [−2, + 2], *ec* = [−1.5, + 1.5], *u* = [−1, 1].

According to formula (2), the values of *q*K⁢e,K⁢e⁢c,K⁢u are: $K\,e=3,K\,e\,c=3,K\,u=\frac{1}{6}.$

### Bioaugmentation

In order to further improve the removal efficiency of waste water, physical and chemical methods and biological processes are generally used to improve the removal efficiency. Activated carbon adsorption, because of its large adsorption capacity and wide application range, is widely used in waste water and drinking water treatment. Activated carbon-activated sludge process, due to the simultaneous physical adsorption and biodegradation, further improves the treatment effect of the system.

#### Source and Characteristics of Sewage

The waste water studied was taken from the actual industrial waste water of a comprehensive waste water treatment plant in a chemical industry park. There are more than 50 different types of chemical enterprises in the industrial park, such as pharmaceutical, petrochemical, electronics, machinery manufacturing, daily chemicals, chemical reagents and so on. The waste water produced by these enterprises is discharged into the pipe network after preliminary treatment, and then collected into the comprehensive waste water treatment plant, and finally discharged into the polluted river after treatment by the waste water treatment plant. The waste water discharged into the pipe network after preliminary treatment by chemical enterprises is comprehensive chemical waste water, which has the characteristics of high pollutant concentration, poor biodegradability, toxicity and inhibition.

#### Test Equipment and Operating Conditions

The research on the removal effect and law of organic matter by adding powdered activated carbon (PAC) into activated sludge system is realized by three groups of SBR reactors, namely activated carbon-activated sludge SBR system (PAC-AS), activated sludge SBR system (AS) and activated carbon SBR system (PAC). The effective volume of each reactor is 2 L, and it runs for 24 h in each cycle. The operation mode is as follows: 1.2 l (15 min) water is fed; Anaerobic stirring (6 h); Aerobic aeration (14 h); Sedimentation (2 h); Drainage 1.2 l (15 min); and Idle (1.5 h).

#### Experimental Method

##### Microbic growth curve

Under aseptic operation conditions, EM bacteria, PP bacteria, and sukehan biological bacteria were mixed with glucose and distilled water according to the ratio of 1:1:100. 200 mL of the mixed solution was sealed in a 250 mL conical flask, and was placed in a shaking table at 25.0°C for shaking culture. Then, 4 mL of bacterial liquid is taken every 2 h, and the OD600 value is measured after dilution. If the OD600 value is within the confidence interval of 0.2–0.8, the final value is measured as x dilution multiple, and if the measured value is not within the interval, the dilution multiple is adjusted for re-measurement.

##### Activation method of microbial agent

According to the mass ratio of 1:9, brown sugar and water were mixed into the culture solution, PP and EM bacteria were inoculated into the brown sugar solution according to the inoculation amount of 10%, then mixed and shaken evenly, and then cultured in a water bath at a constant temperature of 30°C in a shaker at 120 r/min, and centrifuged at 10,000 r/min for 10 min to obtain the bacterial cake. Dilute the bacterial cake with distilled water to prepare bacterial suspension with OD of about 0.2. All centrifuge tubes were sterilized by high pressure steam (121°C, 30 min) before use. The following microbial agents have been activated.

##### Addition of strains

Take a reactor with a volume of 4.5 L and an effective volume of 3 L, clean and autoclave the reactor before use, inoculate the bacterial suspension prepared above into the experimental sewage according to the proportion in Table 1 with an inoculation amount of 10%, set the dissolved oxygen at 3.0 mg/L, react at 30°C for 48 h, take the mud-water mixed sample from the top with a syringe every 3 h, and measure the chroma, viscosity, and lignin after centrifugal separation.

**TABLE 1:** Operation of simulated waste water treatment by biological enhancement.

**Number**	**Reinforcer**	**Water penetration COD (mg/L)**	**Refractory substance COD (mg/L)**	**Effluent COD (mg/L)**	**COD removal rate of refractory substances (%)**	**MLVSS (g/L)**	**U_s_ (kg/kg⋅d)**	**Dissolved oxygen (mg/L)**
1	20% glucose + 0.25 mg/L Fe^3+^	456.33	387.01	48.36	88.01	2.6	0.55	2.1

2	20% rice washing water + 0.25 mg/L Fe^3+^	458.01	379.05	25.58	93.14	2.4	0.52	2.8

3	Contrast	389.36	381.25	55.33	86.33	1.7	0.56	2.4

## Experimental Analysis and Discussion

### Bioenhanced Degradation of Toxic and Refractory Organic Waste Water

The activated sludge is put into a dynamic simulation reactor, and the sludge is cultured and domesticated with nutrients and toxic and refractory organic waste water until it matures. Toxic and refractory organic waste water is prepared from phenol, pyridine and naphthalene, in which pyridine concentration is 40 mg/L and naphthalene concentration is 30 mg/L. Co-metabolism primary matrix is added according to the experimental scheme. Measure COD concentration, MLVSS and dissolved oxygen concentration of inlet and outlet water regularly after the system runs stably. The test results are discussed below.

**FIGURE 4:**
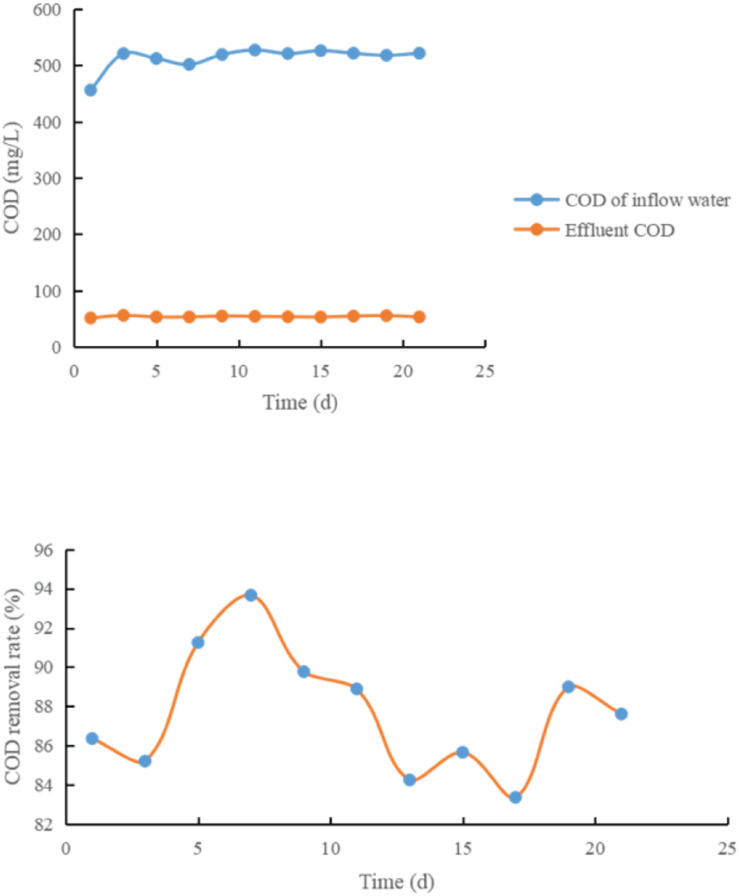
Effect of adding glucose on COD removal rate.

**FIGURE 5:**
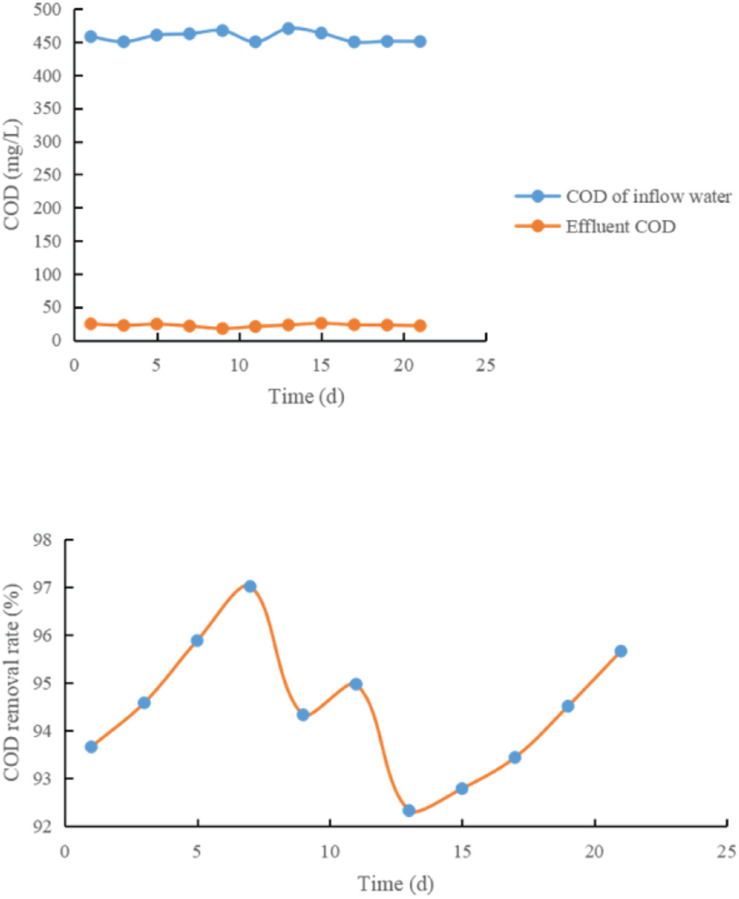
Effect of adding rice washing water on COD removal rate.

**FIGURE 6:**
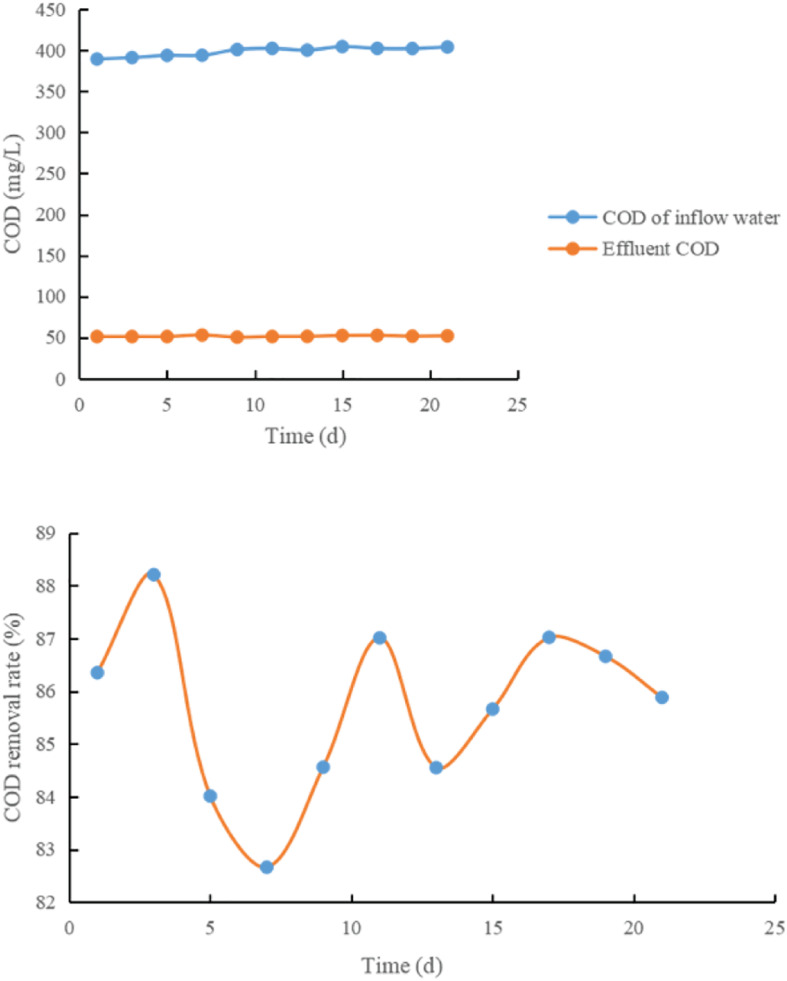
Change curve of COD removal rate of control system.

The test results are shown in Figures 4–6 and Table 1.

It can be seen from Figures 4–6 and Table 1:

(1) Each treatment system has a high COD removal rate for toxic and refractory organic waste water, and the average COD removal rate in 21 days is above 85%, and the operation is stable. The COD removal rate of treatment system 3 is between 83.21 and 86.15%, and the average COD removal rate is 86.33%; 20% glucose was added in the treatment system 1, and the removal rate of COD of refractory substances was 85.33–89.21%, with an average of 88.01%. When 20% rice washing water is added into the treatment system 2, the COD removal rate of refractory substances is between 91.9 and 95.7%. The average is 93.14.

From the above data, it can be seen that adding co-metabolizing primary matrix can improve the COD removal rate of coking waste water in biological treatment system, which may be because the primary matrix promotes the growth of co-metabolizing degradation bacteria and improves the co-metabolizing degradation efficiency of refractory substances.

(2) When rice washing water was used as the primary matrix, the COD removal rate of refractory substances increased by 8.36% compared with the control system, while when glucose was used as the primary matrix, the COD removal rate only increased by 1.87%, indicating that rice washing water was better than glucose in co-metabolism primary matrix.

### Analysis of Removal Characteristics of Organic Matter in Waste Water by Adding PAC

#### Removal Characteristics of Organic Compounds With Different Molecular Weights by Adding PAC

**FIGURE 7:**
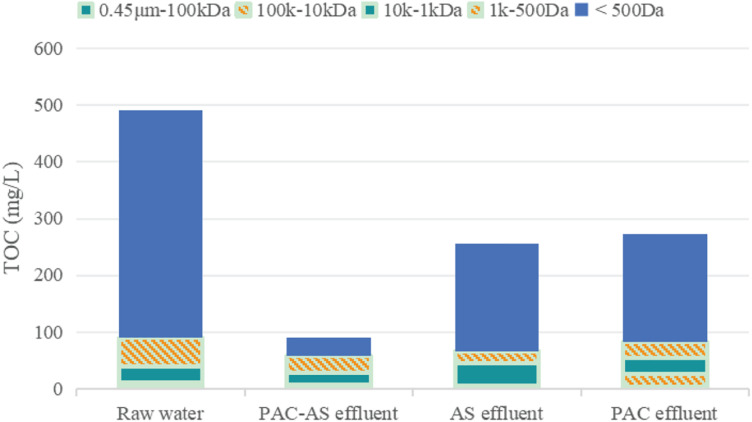
Comparison of molecular weight distribution of organic matter in waste water before and after treatment.

Comparison of molecular weight distribution changes of organic matter in waste water before and after being treated by PAC-AS, AS, and PAC system can reveal the removal rule of organic matter with different molecular weights in waste water by PAC-AS system, as shown in Figure 7. Among them, PAC-AS, AS, and PAC systems are running continuously, and PAC is added continuously. PAC dosing conditions: the initial concentration of PAC is 2 g/l; PAC continuous dosage is 0.25 g/L (0.25 gPAC/L per 4 days) every 4 days. Under this condition, the system can maintain a stable removal effect.

As can be seen from Figure 7:

The AS system has obvious removal effect on organic matters with molecular weights less than 500 Da and 1 k–500 Da, slightly removes organic matters with molecular weights of 0.45 μm–100 kDa, and does not remove organic matters with molecular weights of 10 k–1 kDa and 100 k–10 kDa obviously. Therefore, activated sludge can remove biodegradable parts of organic matters with molecular weights less than 500 Da, 1 k–500 Da, and 0.45 μm–100 kDa, while organic matters with molecular weights of 10 k–1 kDa and 100 k–10 kDa are mainly difficult to biodegrade, so the removal is not obvious.

PAC system has obvious removal effect on organic matter with molecular weight less than 500 Da, and slightly removes organic matter with molecular weights of 1 k–500 Da and 10 k–1 kDa, but has no obvious removal effect on organic matter with molecular weights of 100 k–10 kDa and 0.45 μm–100 kDa. It shows that PAC has a good adsorption effect on small molecular weight organic matter, but with the increase of molecular weight of organic matter, the adsorption effect of PAC decreases gradually, and it has no adsorption effect on large molecular weight organic matter.

Powdered activated carbon-activated sludge SBR system system is obviously superior to AS and PAC system in removing organic matter with molecular weight less than 500 Da, indicating that PAC-AS system is superior to the combined action of PAC adsorption and biodegradation, which makes the removal of organic matter in this molecular weight range show the additive action of PAC adsorption and biodegradation.

#### Removal Characteristics of Different Hydrophilic and Hydrophobic Organic Compounds by Adding PAC

**FIGURE 8:**
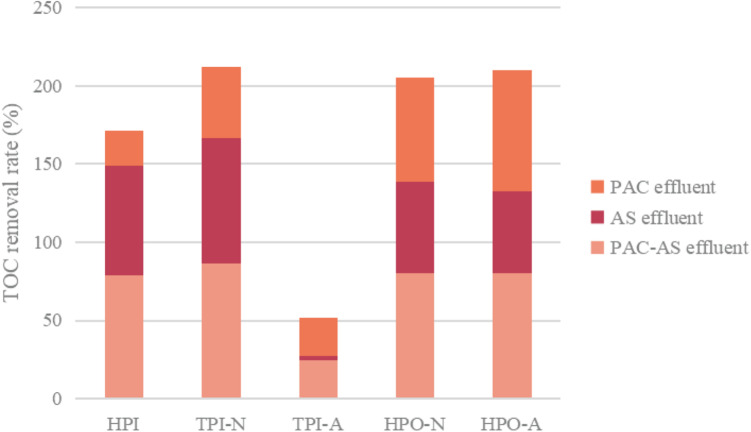
Changes of hydrophilicity and hydrophobicity of organic matter before and after waste water treatment.

The analysis of changes in hydrophilicity and hydrophobicity of organic matter before and after waste water treatment can further reveal the removal rule of organic matter after adding PAC into biological system, as shown in Figure 8 and Table 2.

**TABLE 2:** Removal effect of organic matter in waste water by classification of hydrophilicity and hydrophobicity.

**Classify**	**PAC-AS system**	**AS system**	**PAC system**
**HPO-A**	**++**	**++**	**++**

HPO-N	++	+	++

TPI-A	++	-	+

TPI-N	+	+	+

HPI	++	++	+

*Note “++” means that most of them are removed; “+” means part is removed. “-” means difficult to remove.*

Comparing the raw water with the effluent of AS system, it can be seen that the biodegradable part of each hydrophilic and hydrophobic component can be removed by AS system to a certain extent, and the order of removal rate of each hydrophilic and hydrophobic component from large to small is as follows: TPI-N (73%) > HPI (60%) > HPO-N (55%) > HPO-A (49%) > TPI-A (<2%), That is, TPI-N and HPI are easy to biodegrade, followed by HPO-N and HPO-A, while TPI-A is difficult to biodegrade. It can be seen that hydrophilic components in organic matter are easily degraded and removed by activated sludge, while hydrophobic organic matter is difficult to be degraded by microorganisms. Therefore, the effluent from AS system is mainly hydrophobic organic matter.

Comparing the analysis results of hydrophilic and hydrophobic properties of raw water and PAC system effluent, it can be seen that the removal rate of hydrophilic and hydrophobic components in descending order is HPO-A (72.33%) > HPO-N (62.01%) > TPI-N (48.77%) > TPI-A (26.01%) > HPI (22.01%). It can be seen that PAC has strong adsorption capacity for hydrophobic organic matter (HPO-A and HPO-N), but poor adsorption capacity for hydrophilic organic matter (HPI). Therefore, the effluent from PAC system is mainly HPI component. This is similar to the results obtained in literature (Ran and Li, 2020; Wang et al., 2020), that is, activated carbon mainly adsorbs hydrophobic organic matters, while activated sludge mainly removes hydrophilic organic matters.

After the waste water is treated by PAC-AS system, the order of removal rate of hydrophilic and hydrophobic components is TPI-N (82.01%) > HPO-A (77.86%) > HPO-N (74.33%) > HPI (68.21%) > TPI-A (29.04%) It can be seen that all the components are well removed, and the removal effect is better than that of AS and PAC systems. This is because PAC adsorption and biodegradation can complement each other’s deficiencies in the removal of aqueous organic matter from different relatives, and the two removal effects are additive (Zhang, 2020). For TPI-A component, this part of organic matter is mainly difficult to biodegrade, so in PAC-AS system, the organic matter of this component is mainly removed by PAC adsorption.

### Simulation Result Analysis of Waste Water Treatment System

The optimized adaptive fuzzy neural network control is applied to the established simplified model of waste water treatment. A given dissolved oxygen concentration is 2.0 mg/L, that is, a step signal with a value of 2 is added to the input of the control system, and an interference signal is added to the control model, and simulation experiments are carried out.

**FIGURE 9:**
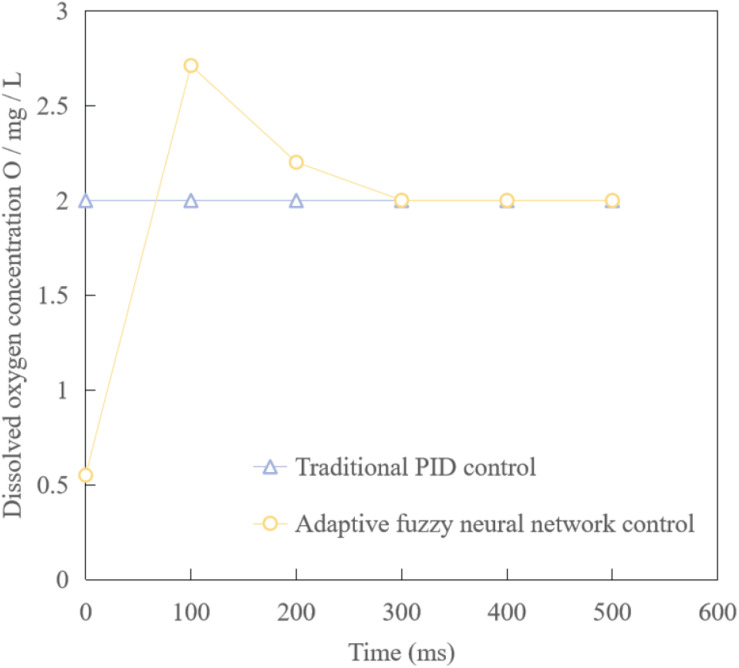
Output control curve of dissolved oxygen concentration.

The control method is applied to the simplified mathematical model formula (1), that is, when the substrate concentration is high and the growth rate of microorganisms is fast, the simulation experiment is carried out, in which each coefficient in the dynamic parameter value of the state equation is uncertain and bounded, and the simulation result is shown in Figure 9. We can see that the controlled object can quickly reach the set value, and has strong anti-interference and the simulation result is stable.

**FIGURE 10:**
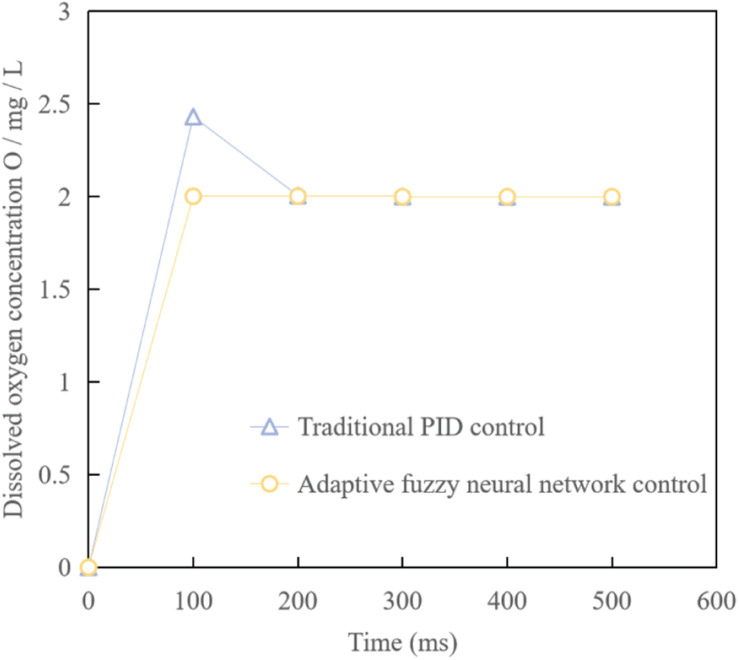
Output control curve of dissolved oxygen concentration.

In the practical application of waste water treatment, traditional control methods (such as PID control) are still adopted in most automatic control links at present, so taking simplified model formula (1) as an example, comparing the control method in this paper with the traditional control method, the simulation results can be obtained as shown in Figure 10. It can be seen from the figure that the optimized adaptive fuzzy neural network control can achieve the control requirements faster than the traditional control method.

## Conclusion

In this paper, aiming at the time-varying and unstable system in the treatment process of toxic and refractory organic waste water, taking the aeration tank of the toxic and refractory organic waste water treatment system as the research object, the architecture of MAS distributed intelligent control waste water treatment system is determined from two aspects of hierarchical structure and model structure, and the system is divided into two parts: control decision system and treatment control system. By comparing the treatment effects of PAC-AS, AS, and PAC systems, the law and characteristics of organic removal after adding PAC into biological system are investigated, and the conclusions are as follows:

(1) Adding co-metabolism primary matrix plays an important role in improving COD removal rate of toxic and refractory organic waste water. The effect of rice washing water as primary substrate is better than that of glucose, and its average COD removal rate is increased by 8.36%, while the average COD removal rate of glucose dosing system is only increased by 1.87%.

(2) According to the classification and analysis of hydrophilicity and hydrophobicity of organic matter before and after waste water treatment, PAC mainly removes hydrophobic organic matter, while activated sludge mainly removes hydrophilic and weakly hydrophobic neutral organic matter. In PAC-AS system, the organic matter of each hydrophilic and hydrophobic component can be removed by microbial degradation and PAC adsorption, so the removal effect of organic matter of each component is further improved.
(3) Compared with the traditional control method, it shows that the fuzzy neural network controller with optimized parameters can achieve better stability when applied to this system, which further shows that the fuzzy neural network control has strong robustness.

## Data Availability Statement

The original contributions presented in the study are included in the article/supplementary material, further inquiries can be directed to the corresponding author.

## Author Contributions

JY conceptualization, investigation, methodology, validation, software, formal analysis, data curation, writing–original draft, and project administration. JJ methodology, writing–review and editing, formal analysis, validation, and funding acquisition. WX resources, writing–review and editing, and language. LW visualization, investigation, writing–review and editing, and formal analysis. JL visualization and investigation. All authors contributed to the article and approved the submitted version.

## Conflict of Interest

The authors declare that the research was conducted in the absence of any commercial or financial relationships that could be construed as a potential conflict of interest.
